# Platelets Promote *Brucella abortus* Monocyte Invasion by Establishing Complexes With Monocytes

**DOI:** 10.3389/fimmu.2018.01000

**Published:** 2018-05-07

**Authors:** Aldana Trotta, Lis N. Velásquez, M. Ayelén Milillo, M. Victoria Delpino, Ana M. Rodríguez, Verónica I. Landoni, Guillermo H. Giambartolomei, Roberto G. Pozner, Paula Barrionuevo

**Affiliations:** ^1^Instituto de Medicina Experimental (IMEX), CONICET, Academia Nacional de Medicina, Buenos Aires, Argentina; ^2^Instituto de Inmunología, Genética y Metabolismo (INIGEM), CONICET, Universidad de Buenos Aires, Buenos Aires, Argentina

**Keywords:** platelets, monocytes/macrophages, *Brucella abortus*, complexes, early infection, brucellosis

## Abstract

Brucellosis is an infectious disease elicited by bacteria of the genus *Brucella*. Platelets have been extensively described as mediators of hemostasis and responsible for maintaining vascular integrity. Nevertheless, they have been recently involved in the modulation of innate and adaptive immune responses. Although many interactions have been described between *Brucella abortus* and monocytes/macrophages, the role of platelets during monocyte/macrophage infection by these bacteria remained unknown. The aim of this study was to investigate the role of platelets in the immune response against *B. abortus*. We first focused on the possible interactions between *B. abortus* and platelets. Bacteria were able to directly interact with platelets. Moreover, this interaction triggered platelet activation, measured as fibrinogen binding and P-selectin expression. We further investigated whether platelets were involved in *Brucella*-mediated monocyte/macrophage early infection. The presence of platelets promoted the invasion of monocytes/macrophages by *B. abortus*. Moreover, platelets established complexes with infected monocytes/macrophages as a result of a carrier function elicited by platelets. We also evaluated the ability of platelets to modulate functional aspects of monocytes in the context of the infection. The presence of platelets during monocyte infection enhanced IL-1β, TNF-α, IL-8, and MCP-1 secretion while it inhibited the secretion of IL-10. At the same time, platelets increased the expression of CD54 (ICAM-1) and CD40. Furthermore, we showed that soluble factors released by *B. abortus*-activated platelets, such as soluble CD40L, platelet factor 4, platelet-activating factor, and thromboxane A_2_, were involved in CD54 induction. Overall, our results indicate that platelets can directly sense and react to *B. abortus* presence and modulate *B. abortus*-mediated infection of monocytes/macrophages increasing their pro-inflammatory capacity, which could promote the resolution of the infection.

## Introduction

*Brucella abortus* is one of the etiological agents of brucellosis, a worldwide zoonotic disease. Human brucellosis has a wide clinical spectrum and is characterized by its tendency to chronicity, associated with successive relapses from acute episodes or focal manifestations (1–3). Brucellosis patients may present diverse hematological alterations, from anemia and leukopenia up to severe hemostatic disorders. Moreover, a reduced platelet count in the bloodstream, i.e., thrombocytopenia, is frequently observed and might be severe (4–6). Although thrombocytopenia is associated with a higher rate of relapses (5, 7), the etiology of this disorder has not been elucidated yet. Even more, the role of platelets in the context of *Brucella* infection remains completely unknown.

Bacteria from the genus *Brucella* are Gram-negative microorganisms able to survive and reproduce inside phagocytic cells as facultative intracellular pathogens. Once inside their host, these bacteria have an extracellular dissemination phase before reaching the macrophage, their preferential intracellular niche. Platelets, along with neutrophils and monocytes, are one of the first cells to encounter bacteria during this extracellular phase (2, 3). Then, bacteria are phagocyted by neutrophils and monocytes, and transported by the bloodstream to the liver’s sinusoids, spleen, bone marrow, and lymph nodes, where they are able to multiply and survive inside macrophages.

*Brucella abortus* infection activates both innate and adaptive immune responses and generates a pro-inflammatory environment that favors the differentiation of CD4^+^ T cells toward a Th1 phenotype (8–11). However, *B. abortus* is able to persist inside the macrophages evading the host immune response. This ability determines the disease progression, which includes its tendency to recidivism and evolution into chronic forms (1–3).

Platelets have been thoroughly described as hemostatic mediators and responsible for maintaining vascular integrity (12). Nevertheless, recent studies have demonstrated that platelets also have an important role in the modulation of innate and adaptive immune responses (12–15). As well as the receptors for thrombotic stimuli as collagen (GPVI), adenosine-di-phosphate (P2Y1/12), and thrombin (PAR1/4), platelets have a wide spectrum of receptors for pathogenic and immunological molecules, similar to professional phagocytes (16). Toll-like receptors (TLRs), receptors for complement and for the Fc portion of the IgG (FcγRII), are included within this group of receptors (17, 18). Through these receptors, platelets can become activated in response to different microorganisms and secrete a vast amount of products contained in their granules (19–21). Among these products are soluble CD40L (sCD40L), platelet-activating factor (PAF), thromboxane A_2_ (TXA_2_), and several pro-inflammatory cytokines and immunomodulatory chemokines such as platelet factor 4 (PF4), RANTES, and CXCL7 (22, 23). Moreover, it has been demonstrated that platelets are able to produce and secrete antimicrobial molecules, including defensins and thrombocidins (24–27).

In the past few years, there have been significant advances in the study of the interactions of platelets with several infectious agents (12–14). Beyond their ability to respond to different pathogens and bactericide activity, platelets can also lead the innate and adaptive immune responses through their interaction with several leukocyte populations, particularly with neutrophils and monocytes (28–30). Upon activation, platelets expose P-selectin on their surface, which facilitates the interaction with neutrophils and monocytes, and allows the formation of platelet–leukocyte complexes or aggregates (31). In the case of neutrophils, it has been described that this particular interaction leads to neutrophil extracellular traps (NETs) formation, which contributes to the restraint of bacterial infection (24, 32). Regarding platelet–monocyte complexes, it has been reported that platelets can modulate the secretion of several monocyte cytokines, such as IL-10 and TNF-α (13, 33) and the surface expression of co-stimulatory molecules in response to bacterial stimulation (13). Overall, these responses might facilitate the control of the infection, but can also contribute to the pathogenesis of the infectious disease (12, 14). Thus, platelets can play either a beneficial or a detrimental role during infection elicited by different pathogens.

Important progress has been made in the study of platelets interactions with infectious agents and the modulation of immune responses mediated by platelets. Despite this, whether platelets interact with bacteria of genus *Brucella* and/or are able to modulate any aspect of the *Brucella*-elicited immune response still remains unknown. Therefore, the aim of this work was to elucidate the role of platelets in the immune response against *B. abortus*. In this study, we first focused on the possible interaction between *B. abortus* and platelets. Once this phenomenon was corroborated, we investigated the role of platelets in the development of monocyte/macrophage early infection by *B. abortus*. Here, we present the results of this study.

## Materials and Methods

### Ethics Statement

Human platelets and monocytes were isolated exclusively from healthy adult blood donors in agreement with the guidelines of the Ethical Committee of the IMEX Institute (protocol number: 20160518-M). All adult blood donors provided their informed consent prior to the study in accordance with the Declaration of Helsinki (2013) of the World Medical Association.

### Bacteria

*Brucella abortus* S2308 and green fluorescence protein (GFP)-S2308 (34) were cultured in tryptose-soy agar supplemented with yeast extract (Merck). The number of bacteria on stationary-phase cultures was determined by comparing the OD at 600 nm to a standard curve. All live *Brucella* manipulations were performed in biosafety level 3 facilities, located at the Instituto de Investigaciones Biomédicas en Retrovirus y SIDA (Buenos Aires, Argentina).

### Regents

Recombinant PF4 was purchased from PeproTech. Both recombinant CD40L and the anti-CD40L neutralizing antibody were purchased from BioLegend. Acetylsalicylic Acid (Aspirin) was purchased from Sigma-Aldrich. Anti-PF4 neutralizing antibody was purchased from Abcam.

### Cells and Media

All experiments were performed at 37°C in 5% CO_2_ atmosphere and standard medium composed of RPMI-1640 supplemented with 25 mM Hepes, 2 mM l-glutamine, 10% heat-inactivated fetal bovine serum (Gibco), 100 U of penicillin/ml, and 100 µg of streptomycin/ml. THP-1 cells were obtained from the American Type Culture Collection (Manassas, VA, USA) and cultured as previously described (35). To induce maturation, cells were cultured in 0.05 µM 1,25-dihydroxyvitamin D_3_ (EMD Millipore) for 72 h. Peripheral blood mononuclear cells (PBMCs) were obtained by Ficoll-Hypaque (GE Healthcare) gradient centrifugation from human blood collected from healthy adult individuals. Monocytes were then purified from PBMCs by Percoll (GE Healthcare) gradient and resuspended in standard medium. Purity of the isolated CD14^+^ monocytes was more than 80% as determined by flow cytometry. Viability of cells was more than 95% in all the experiments as measured by trypan blue exclusion test.

### Platelets

Washed platelets (WPs) were obtained from human whole blood from healthy adult donors. Blood samples were directly collected into plastic tubes containing 3.8% sodium citrate (10:1) (Merck). Platelet-rich plasma (PRP) was obtained by blood sample centrifugation. To avoid leukocyte contamination, only the top 75% of the PRP was collected. The PRP was centrifuged in presence of 75 nM prostaglandin I_2_ (PGI_2_) (Cayman Chemical), and platelets were then washed with RPMI-1640 medium. Finally, WPs were resuspended in RPMI-1640 medium.

### Fibrinogen-Binding Assay

Platelets were incubated with *B. abortus* (PLT:*Ba* ratio of 1:10) or 0.05 U of thrombin/ml for 10 min at room temperature in presence of Alexa 488-labeled fibrinogen. Then, platelets were fixed in a 4% paraformaldehyde solution, and fibrinogen binding was evaluated by flow cytometry.

### P-Selectin Expression Assay

Platelets were incubated with *B. abortus* (PLT:*Ba* ratio of 1:10) or 0.05 U of thrombin/ml for 10 min at room temperature. Platelets were then stained with FITC-labeled anti-human P-selectin antibody (BD Biosciences) or its isotype control and the expression of surface P-selectin was evaluated by flow cytometry.

### *In Vitro* Infection

THP-1 cells at a concentration of 0.5 × 10^6^/ml were infected in round-bottom polypropylene tubes (Falcon) with a multiplicity of infection (MOI) of 100 of *B. abortus* S2308 or GFP-S2308, in presence or absence of platelets (THP-1:PLT ratio 1:100). All infections were performed for 2 or 4 h in standard medium containing no antibiotics. In all cases, cells were then extensively washed to remove uninternalized bacteria, and infected cells were maintained in culture in medium supplemented with 100 µg of gentamicin/ml and 50 µg of streptomycin/ml. At different times post-infection, supernatants were collected, filtered, and stored at −70°C for later determination by Enzyme Linked ImmunoSorbent Assay (ELISA). In another set of experiments, the cells were equally infected with *B. abortus* in presence or absence of platelets, and the expression of surface markers was evaluated by flow cytometry.

### Platelet Supernatants for Monocyte Stimulation

*Brucella abortus* (1 × 10^7^/ml) were incubated with platelets (PLT:*Ba* ratio of 1:1) for 4 h. Then, the supernatants were collected, sterilized by filtration, ultracentrifuged, and stored at −70°C.

### Colony-Forming Units (CFU) Count

#### Platelets Infection Assay

Platelets were infected with *B. abortus* at different concentrations for 2 h in standard medium containing no antibiotics. In all cases, platelets were then treated with 100 µg of gentamicin/ml and 50 µg of streptomycin/ml for 30 min to eliminate all uninternalized bacteria. Finally, platelets were lysed with 0.01% v/v Triton X-100 and the lysate was plated in tryptose-soy agar supplemented with yeast extract. The CFU were counted 4 days post-plated.

#### Monocytes Infection Assay

THP-1 cells were infected with *B. abortus* (MOI 100) in presence of platelets at different concentrations (THP-1:PLT ratio of 1:1, 1:10, and 1:100) for 2 h in standard medium containing no antibiotics. In all cases, monocytes were then treated with 100 µg of gentamicin/ml and 50 µg of streptomycin/ml for 30 min to eliminate all uninternalized bacteria and incubated for different times as specified in each figure. Finally, monocytes were lysed with 0.01% v/v Triton X-100, and the lysate was plated in tryptose-soy agar supplemented with yeast extract. The CFU were counted 4 days post-plated.

### Platelet–Monocyte Complexes Quantification

Whole blood of healthy donors was stimulated with *B. abortus* (2 × 10^5^ bacteria/ml) for 30 min. Then, monocytes were stained with a PerCP-labeled anti-CD14 (BioLegend) and platelets with a PE-labeled anti-CD61 antibody (BD Biosciences). Finally, the samples were fixed, and red blood cells were lysed with BD FACS^®^ Lysing Solution (BD Biosciences) and analyzed on a FACSCalibur^®^ flow cytometer (BD Biosciences). Cells from whole blood were plotted on a CD14 vs. SSC dot plot. Then, the CD14^+^ cells were plotted on a CD14 vs. CD61 dot plot. Finally, the presence of platelet–monocyte complexes (CD14^+^CD61^+^) was determined. Data were processed using CellQuest software (BD Bioscience) or FlowJo^®^ 7.6 software (LLC).

### Enzyme Linked ImmunoSorbent Assay

Human TNF-α (BD Bioscience), IL-1β (BioLegend), IL-10 (BioLegend), IL-8 (BioLegend), and MCP-1 (BD Bioscience) concentration was measured in culture supernatants of monocytes, *B. abortus*, and/or platelets by sandwich ELISA, using paired cytokine-specific Abs according to the manufacturer’s instructions.

### Flow Cytometry

THP-1 cells (0.5 × 10^6^/ml) were infected with a multiplicity of infection of *B. abortus* S2308 (MOI 100) in presence or absence of platelets (THP-1:PLT ratio of 1:100) for 4 h. Cells were then washed to remove uninternalized bacteria and cells were stained with PE-labeled anti-human CD54 (clone HA58; BioLegend), anti-human CD40 (clone 5C3; BioLegend) or their isotype-matched control Abs. After labeling, cells were analyzed on a FACSCalibur^®^ flow cytometer (BD Biosciences), and data were processed using CellQuest software (BD Bioscience) or FlowJo^®^ 7.6 software (LLC).

### Confocal Microscopy

#### Platelet–*B. abortus* Interaction

Platelets (5 × 10^6^ platelet/well) were incubated with GFP-*B. abortus* (PLT:*Ba* ratio of 1:1, 1:10, and 1:30) in RPMI medium for 2 h in *chamber-slides* pre-treated with 7.5 ng of Poly l-lysine/ml. Then, cells were fixed with 2% paraformaldehyde and stained with an anti-CD61 Ab (VI-PL2; BD Bioscience) followed by an Alexa 546-labeled secondary Ab (Molecular Probes Life Technologies).

#### Monocyte–*B. abortus*–Platelet Interaction

THP-1 cells (2 × 10^5^ cells/well) were incubated in chamber-slides with 10 ng of PMA/ml for 24 h to promote adherence. Then, cells were treated as indicated in each figure, fixed with 2% paraformaldehyde and permeabilized with 0.1% saponin. The samples were then incubated with an anti-HLA-ABC class I mAb (W6/32) followed by Alexa 633-labeled secondary Ab (Molecular Probes Life Technologies) and an anti-CD61 Ab (VI-PL2; BD Bioscience) followed by an Alexa 546-labeled secondary Ab (Molecular Probes Life Technologies).

In all cases, slides were mounted with PolyMount (Polysciences) and analyzed using a FV-1000 confocal microscope with an oil-immersion Plan Apochromatic 60× NA1.42 objective (Olympus). The obtained images were processed with FIJI software (open source).

### Statistical Analysis

Results were analyzed with one or two-way ANOVA followed by *post hoc* Tukey test using the GraphPad Prism software.

## Results

### *B. abortus* Directly Interacts With Platelets

**Figure 1:**
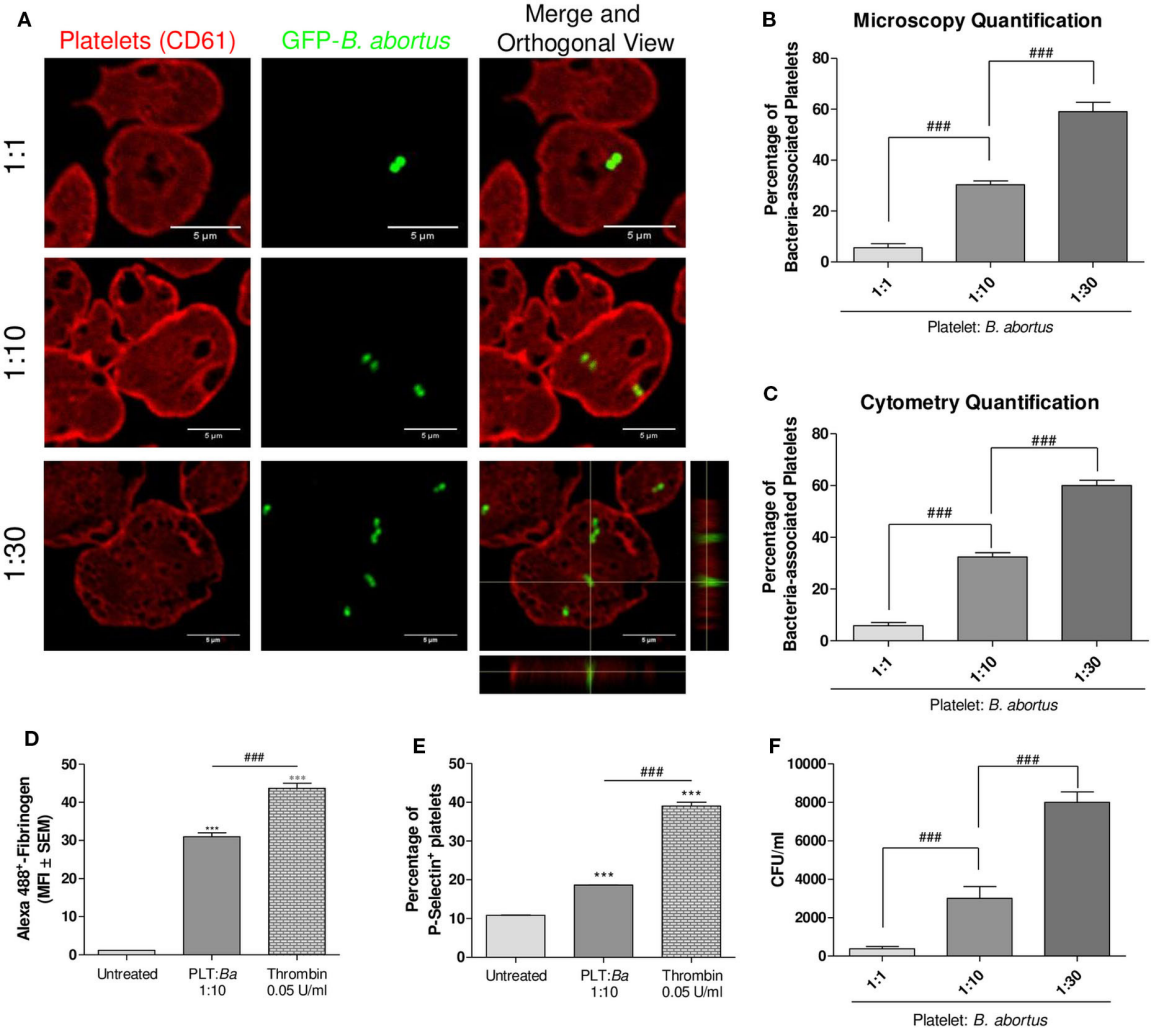
There is a direct interaction between platelets and *Brucella abortus*. **(A)** Confocal micrographs of platelets incubated with green fluorescence protein (GFP)-*B. abortus* at different ratios (Platelet:GFP-*B. abortus* 1:1, 1:10, and 1:30) for 4 h. Platelet population was stained with an anti-human CD61 primary Ab and Alexa 546-labeled secondary Ab (red). **(B)** Quantification of platelet–*B. abortus* interaction by confocal microscopy. The number of platelets counted per experimental group was 200. **(C)** Quantification of platelet–*B. abortus* interaction by flow cytometry. Data are expressed as the percentage of platelets associated with *B. abortus* (GFP-positive platelets) ± SEM of three independent experiments. Platelets were also incubated with *B. abortus* for 30 min and their activation status was measured as Fibrinogen binding **(D)** and P-selectin expression **(E)**. The platelet activator thrombin was used as control. Bars represent the arithmetic means ± SEM of three experiments or the percentage of platelets that express P-selectin. MFI, mean fluorescence intensity. **(F)** Quantification of platelet invasion by *B. abortus*. Data are expressed as colony-forming units (CFU) per ml. ***P < 0.001 vs. untreated; ^###^*P* < 0.001.

We first investigated whether a direct interaction between *B. abortus* and platelets occurs. For this, platelets were co-incubated with GFP-*B. abortus* at different ratios (Platelet:GFP-*B. abortus* 1:1, 1:10, and 1:30) for 4 h. Then, platelets were stained with an anti-CD61 antibody and the platelet–*B. abortus* interaction was quantified by confocal microscopy (Figures 1A,B) and flow cytometry (Figure 1C and gating strategy shown in Figure S1 in Supplementary Material). *B. abortus* was able to directly bind to platelets and this interaction increased in a dose-dependent manner (Figures 1A–C). Taking into account that several bacteria are able to modify platelet function (36), we next evaluated whether the physical interaction with *B. abortus* was able to modulate platelet functional responses. For this, platelets were incubated with *B. abortus* and platelet activation was then evaluated by flow cytometry within the platelet gate determined in the FSC vs. SSC dot plot as showed in Figure S1A in Supplementary Material. *B. abortus* was able to significantly increase both fibrinogen binding and P-selectin expression, though to a lesser extent than the platelet activator thrombin (Figures 1D,E). Next, we wondered whether *B. abortus* was able to invade platelets. For this, platelets were incubated with different multiplicities of infection (MOI) of *B. abortus* for 2 h. Afterward, extracellular bacteria were killed by adding antibiotics, platelets were lysed, and the number of viable intracellular bacteria was determined by plating the lysates on tryptose-soy agar. As shown in Figure 1F, *B. abortus* was able to invade platelets in a dose-dependent manner. Overall, these results demonstrate that *B. abortus* is able to directly interact with and invade platelets, triggering their activation.

### Platelets Promote Monocyte/Macrophage Invasion by *B. abortus*

**Figure 2:**
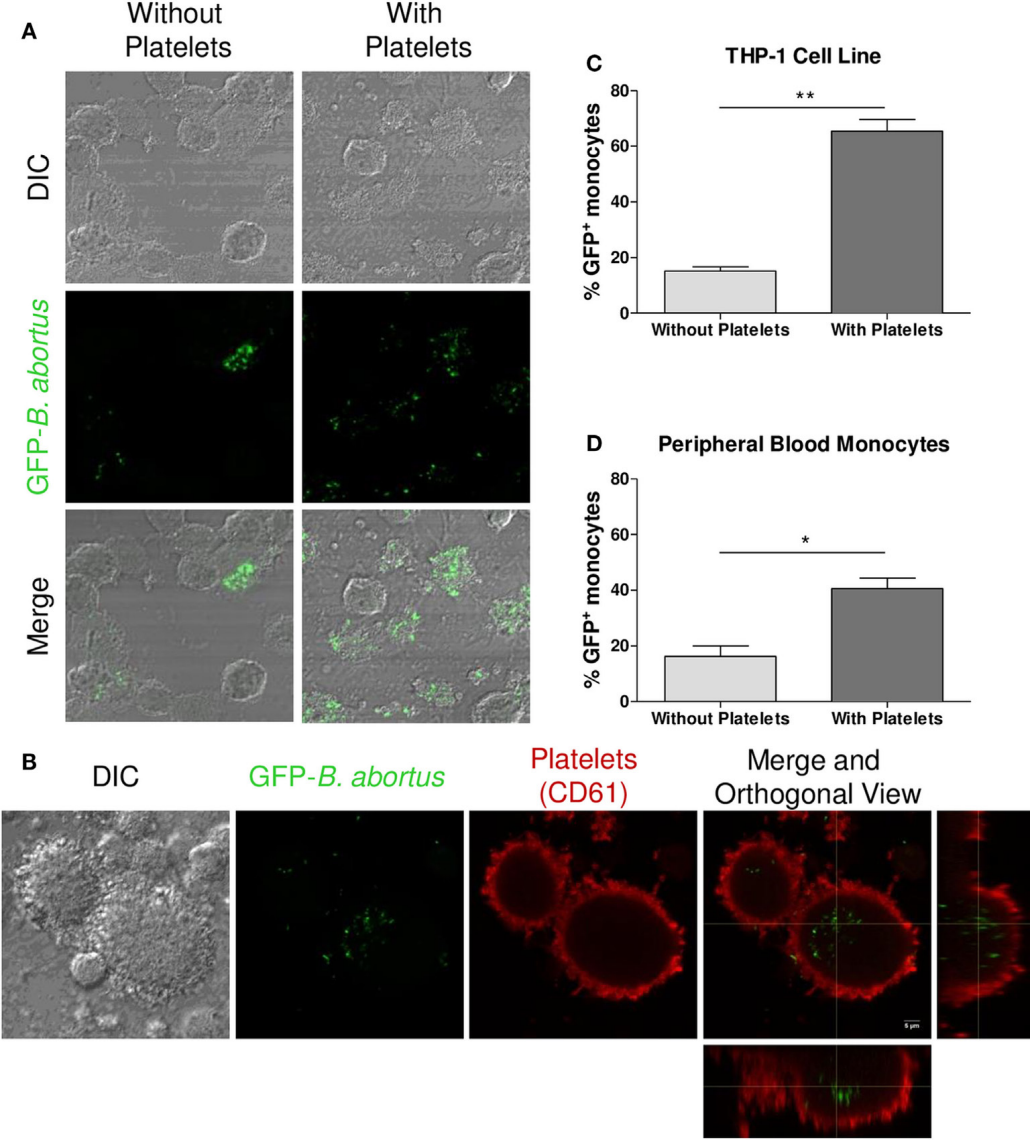
Platelets promote monocyte/macrophage invasion by *Brucella abortus*. **(A)** Confocal micrographs of THP-1 cells infected with green fluorescence protein (GFP)-*B. abortus* in presence or absence of platelets for 4 h. **(B)** Confocal micrographs of THP-1 cells infected with GFP-*B. abortus* in presence of platelets for 4 h. Platelets were stained with an anti-human CD61 primary Ab and Alexa-546-labeled secondary Ab (red). **(C)** Quantification of THP-1 cell invasion by *B. abortus*. **(D)** Quantification of peripheral blood-isolated human monocyte invasion by *B. abortus*. Data are expressed as the percentage of monocytes invaded (GFP-positive monocytes) ± SEM of three independent experiments. The number of cells counted per experimental group was 200. *P < 0.05; **P < 0.01 vs. monocytes without platelets. THP-1:PTL:*Ba* 1:100:100.

Once the interaction between platelets and bacteria was demonstrated, and taking into consideration that monocytes/macrophages are the main niche for *B. abortus* replication, we further investigated the capacity of platelets to modulate the monocytes/macrophages infection mediated by *B. abortus*. For this, THP-1 cells were infected with GFP-*B. abortus* for 4 h, in presence or absence of platelets and THP-1 infection was assessed by confocal microscopy. As shown in Figures 2A–C, the presence of platelets significantly increased the percentage of *B. abortus*-infected THP-1 cells (GFP^+^). This phenomenon was reproduced using human monocytes purified from peripheral blood (Figure 2D). Altogether, these results indicate that the presence of platelets promote the invasion of monocytes/macrophages by *B. abortus* at early times during infection.

### Platelets Establish Complexes With *B. abortus*-Infected Monocytes

**Figure 3:**
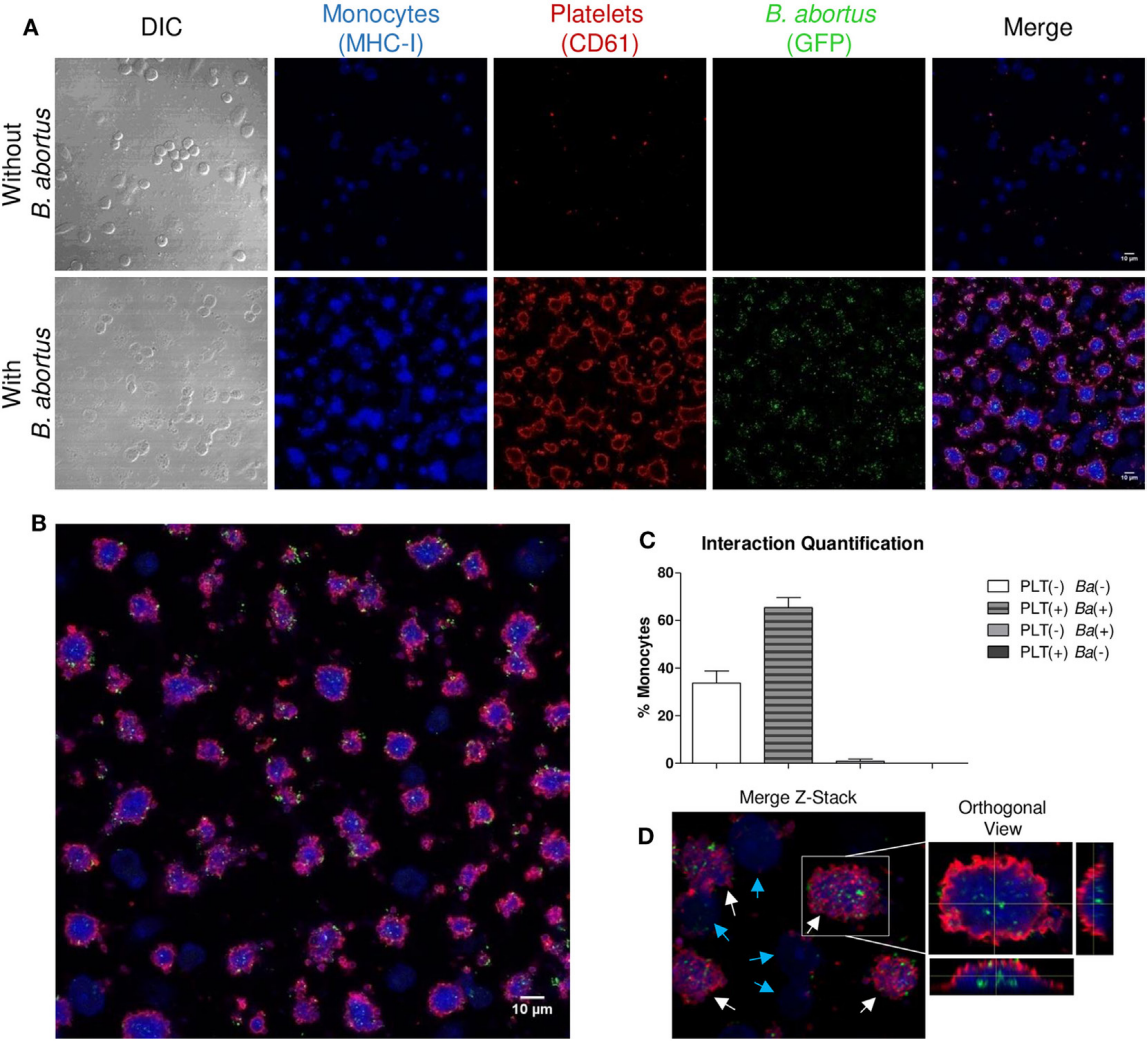
Platelets surround *Brucella abortus*-infected monocytes. **(A)** Confocal micrographs of THP-1 cells infected with green fluorescence protein (GFP)-*B. abortus* in presence or absence of platelets for 4 h. Monocyte population was stained with an anti-human MHC-I primary Ab and Alexa-633-labeled secondary Ab (blue). Platelet population was stained with an anti-human CD61 primary Ab and Alexa-546-labeled secondary Ab (red). **(B)** Confocal panoramic micrograph of THP-1 cells infected with GFP-*B. abortus* in presence of platelets, representative of the micrographs used for the interaction quantification. **(C)** Quantification of the interactions between populations. The monocytes subpopulations were defined as: non-infected and without platelets (double-negatives), infected and associated with platelets (double-positives), only infected (GFP-positives), and only associated with platelets (CD61-positives). Data are expressed as percentage of monocytes ± SEM of three independent experiments. The number of cells counted per experimental group was 200. **(D)** Merge Z-stack and orthogonal view of THP-1 cells infected with GFP-*B. abortus* in presence of platelets for 4 h. Blue arrows indicate non-infected THP-1 cells while white arrows indicate infected THP-1 cells surrounded by platelets.

**Figure 4:**
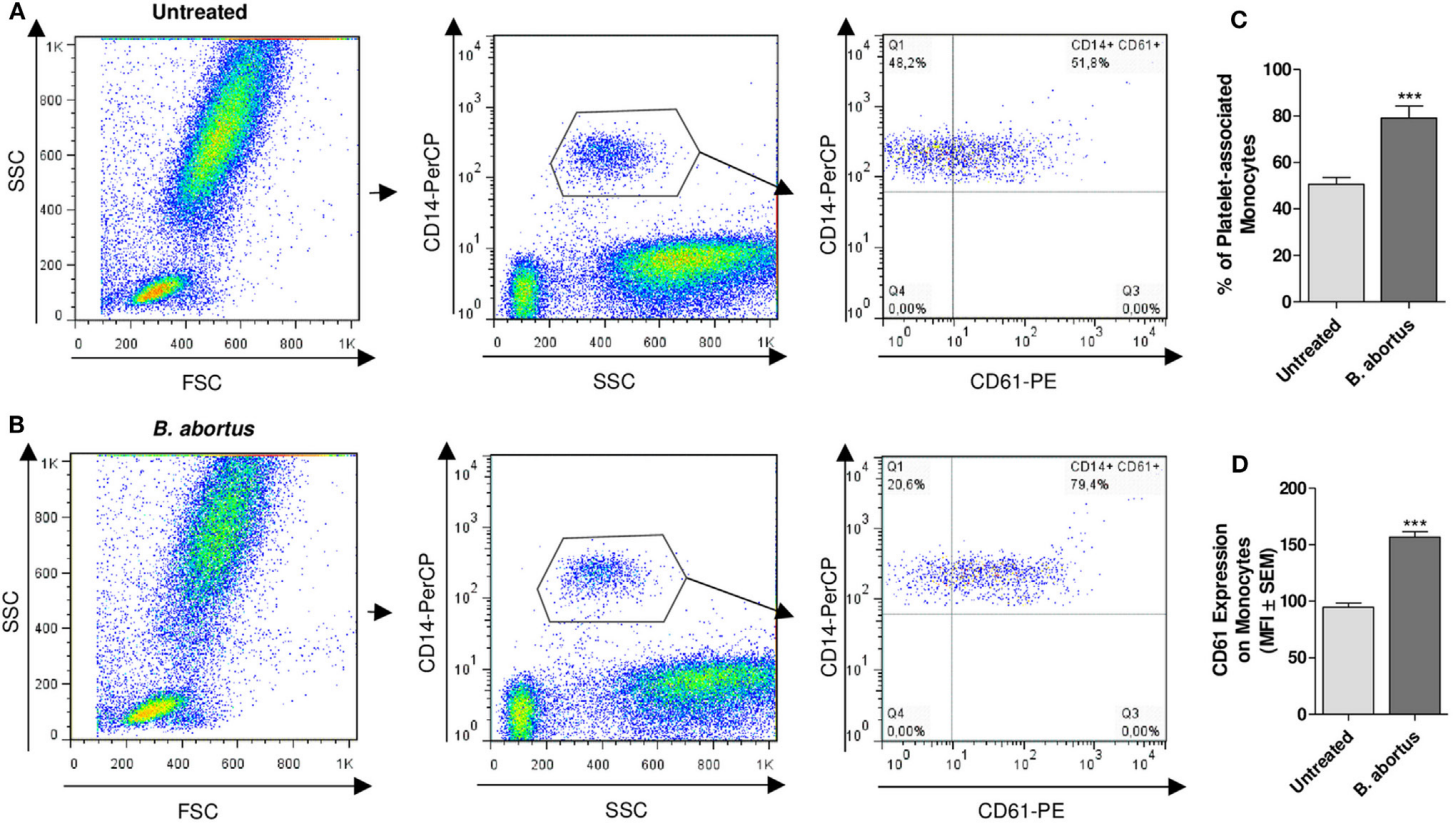
*Brucella abortus* infection promotes platelet–monocyte complexes formation in whole blood. Flow cytometry analysis of whole blood untreated **(A)** or treated with *B. abortus* **(B)** for 30 min. Monocyte population was stained with a PerCP-labeled anti-CD14 Ab and platelet population was stained with a PE-labeled anti-CD61 Ab. Cells were plotted on a CD14 vs. SSC dot plot. Then, the CD14^+^ cells were plotted on a CD14 vs. CD61 dot plot. Finally, the presence of platelet–monocyte complexes (CD14^+^CD61^+^) was determined. **(C)** Quantification of platelet–monocyte complexes within the CD14^+^ gate. Data are expressed as the percentage of monocytes associated with platelets (CD14^+^CD61^+^ cells) ± SEM of three independent experiments. **(D)** CD61 expression in platelet-bearing monocytes (CD14^+^CD61^+^). Bars represent the arithmetic means ± SEM of three experiments. MFI, mean fluorescence intensity. THP-1:PTL:*Ba* 1:100:100. ***P < 0.001 vs. Untreated.

Having demonstrated the interaction between platelets and bacteria and the promotion of monocyte invasion, we wondered how the interactions between these three cell populations were. For this, THP-1 cells were incubated with platelets in a THP-1:Platelet ratio of 1:100, in presence or absence of GFP-*B. abortus* (MOI 100 respect to THP-1) for 4 h. Then, monocytes were stained with anti-MHC-I (blue) and platelets with anti-CD61 (red) antibodies. Finally, samples were assessed by confocal microscopy. Monocytes established complexes with platelets in presence of *B. abortus* (Figure 3A). Interestingly, platelets were associated with monocytes that were infected with *B. abortus* (*Ba*-positive monocytes) (Figures 3B,D). Next, the formation of platelet–monocyte complexes mediated by *B. abortus* was quantified. For this, the number of monocytes per field was quantified and we analyzed which of them were infected (*Ba*-positive), not infected (*Ba*-negative), associated with platelets (PLT-positive) or not associated with platelets (PLT-negative). The percentage of double-negative monocytes (not infected and not associated with platelets), double-positive (infected and associated with platelets), and single-positive (not infected but associated with platelets, or infected but not associated with platelets) was represented. Only *B. abortus*-infected monocytes were able to establish complexes with platelets (Figure 3C). Moreover, as shown in Figure 3D and Video S1 in Supplementary Material, platelets were disposed around the infected monocytes so as to surround them completely. Next, we wondered whether these platelet–monocyte complexes could be established in whole blood. For this, whole blood of healthy donors was incubated with *B. abortus* and then stained with anti-CD14 and anti-CD61 antibodies. Afterward, the presence of platelet–monocyte complexes (CD14^+^CD61^+^) within the CD14^+^ gate was assessed by flow cytometry (Figures 4A,B). As shown in Figures 4A–C, *B. abortus* was able to increase not only the percentage of monocytes bound to platelets (% of CD14^+^CD61^+^ cells) but also the quantity of adhered platelets per monocyte (CD61 expression on cells corresponding to CD14^+^CD61^+^ Quadrant) (Figure 4D). Overall, these results demonstrate that the presence of *B. abortus* promotes the establishment of platelet–monocyte complexes. Interestingly though, platelets exclusively surround monocytes infected with *B. abortus*.

### Platelets Act as Carriers of Bacteria Toward Monocytes

**Figure 5:**
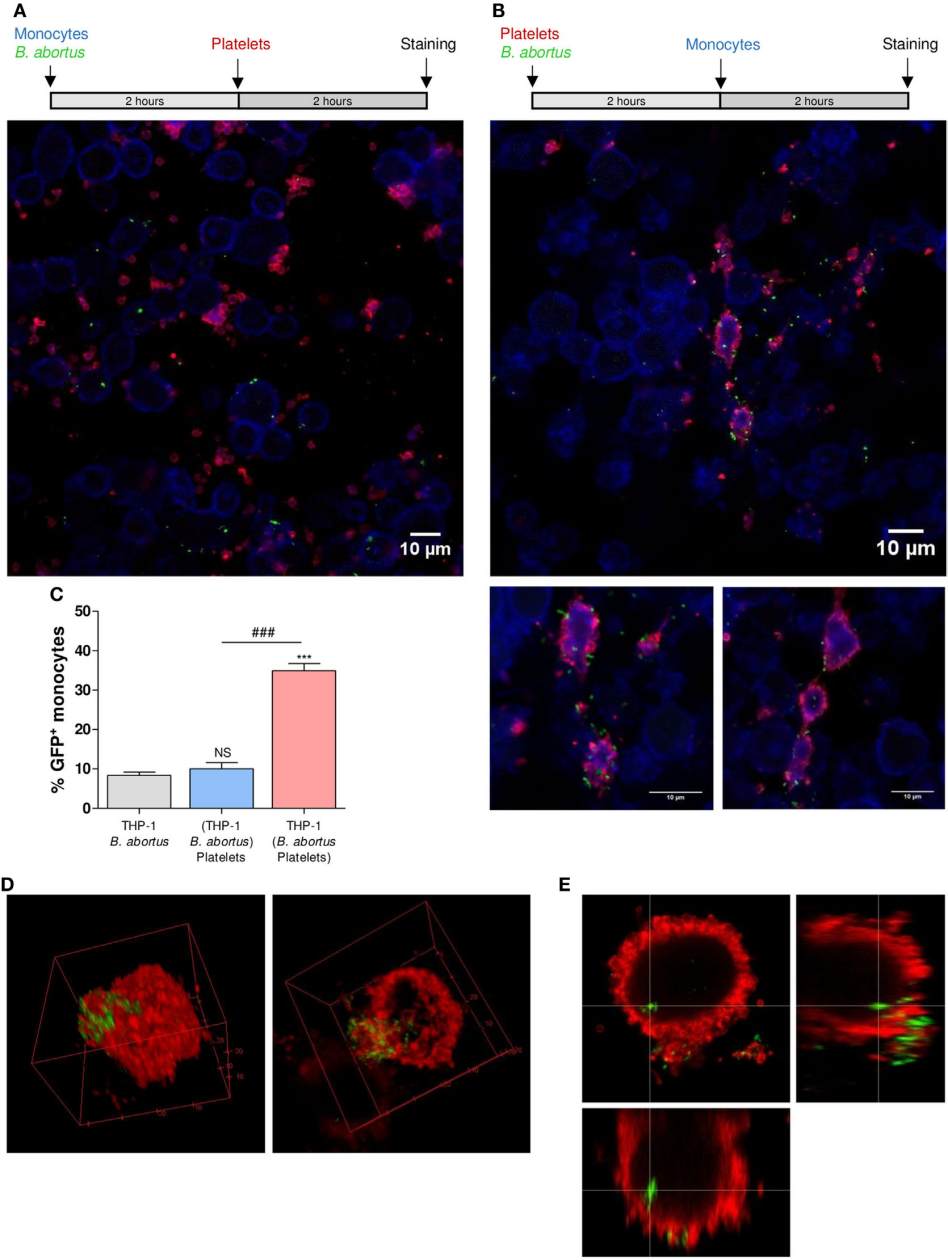
Platelets act as carriers of bacteria toward monocytes. **(A)** Confocal micrographs of THP-1 cells incubated with green fluorescence protein (GFP)-*Brucella abortus* for 2 h and then incubated with platelets for additional 2 h. **(B)** Platelets were incubated with GFP-*B. abortus* for 2 h. THP-1 cells were then incubated with this suspension for additional 2 h. Monocyte population was stained with an anti-human MHC-I primary Ab and Alexa-633-labeled secondary Ab (blue). Platelet population was stained with an anti-human CD61 primary Ab and Alexa-546-labeled secondary Ab (red). Results are representative of three independent experiments. THP-1:PTL:*Ba* 1:100:100. **(C)** Quantification of THP-1 cell invasion by *B. abortus* for three experimental conditions: *B. abortus*-infected monocytes in absence of platelets and experimental conditions shown in panels **(A,B)**. Data are expressed as the percentage of monocytes invaded (GFP-positive monocytes) ± SEM of three independent experiments. The number of cells counted per experimental group was 200. NS, not significant; ***P < 0.001 vs. THP-1 + *B. abortus*; ^###^*P* < 0.001. **(D,E)** Platelets were incubated with GFP-*B. abortus* for 2 h. THP-1 cells were then incubated with this suspension for additional 2 h. Platelets were stained with an anti-human CD61 primary Ab and Alexa-546-labeled secondary Ab (red). 3D reconstruction **(D)**; and Merge Z-stack and orthogonal view **(E)**.

The fact that platelets form complexes with *B. abortus*-infected monocytes led us to postulate two possible hypotheses regarding the mechanism involved in this phenomenon: either (1) infected monocytes increase the expression of some surface molecules or receptors, supporting the specific binding of platelets or (2) activated platelets act as carriers of bacteria, transporting them toward the monocytes and, in this way, promoting monocyte infection. To evaluate the first hypothesis, THP-1 cells were infected with GFP-*B. abortus* (MOI 100) for 2 h. Then, cells were washed and incubated with platelets for additional 2 h. Monocytes incubated for 2 h in absence of *B. abortus* were used as control (data not shown). Finally, the samples were stained with anti-MHC-I (blue) and anti-CD61 (red) antibodies and assessed by confocal microscopy. When monocytes were first infected with *B. abortus* and incubated with platelets afterward, the obtained percentage of infection was similar to the control condition of *B. abortus*-infected monocytes in absence of platelets (Figures 5A,C). Moreover, it was observed that infected monocytes were not surrounded by platelets as in the monocyte–*B. abortus*–platelet co-incubation treatment (Figure 5A). In this case, although some platelets were attached to the surface of infected monocytes, the number and disposition of platelets around monocytes was different from the co-incubation experiments (Figure 5A).

To evaluate the second hypothesis, platelets were incubated with GFP-*B. abortus* for 2 h. Next, THP-1 cells were incubated with *B. abortus*-stimulated platelet suspension for additional 2 h. A suspension of platelets incubated for 2 h in absence of *B. abortus* was used as control (data not shown). Finally, the samples were stained as described before and assessed by confocal microscopy. When monocytes were stimulated with the pre-incubated suspension of platelets and *B. abortus*, the percentage of infection was higher than the experimental condition shown in Figure 5A (Figures 5B,C). In addition, using this experimental approach, *B. abortus*-infected monocytes associated with platelets were observed. Furthermore, platelets completely surrounded the infected monocytes in the same manner as described in the monocyte–*B. abortus*–platelet co-incubation treatment (Figure 5B; Video S2 in Supplementary Material). Supporting the carrier activity of platelets, it was observed the presence of platelets delivering bacteria to monocytes (Figures 5D,E; Video S3 in Supplementary Material). Overall, these results indicate that the infection of monocytes does not induce the formation of platelet–monocyte complexes. Instead, the interaction between platelets and bacteria triggers platelet activation and promote platelets to act as carriers of bacteria toward monocytes. As a result, *B. abortus*-infected monocytes remain associated with the platelets that transported bacteria toward them.

### Platelets Modulate Cytokine and Chemokine Secretion in the Context of *B. abortus* Infection

**Figure 6:**
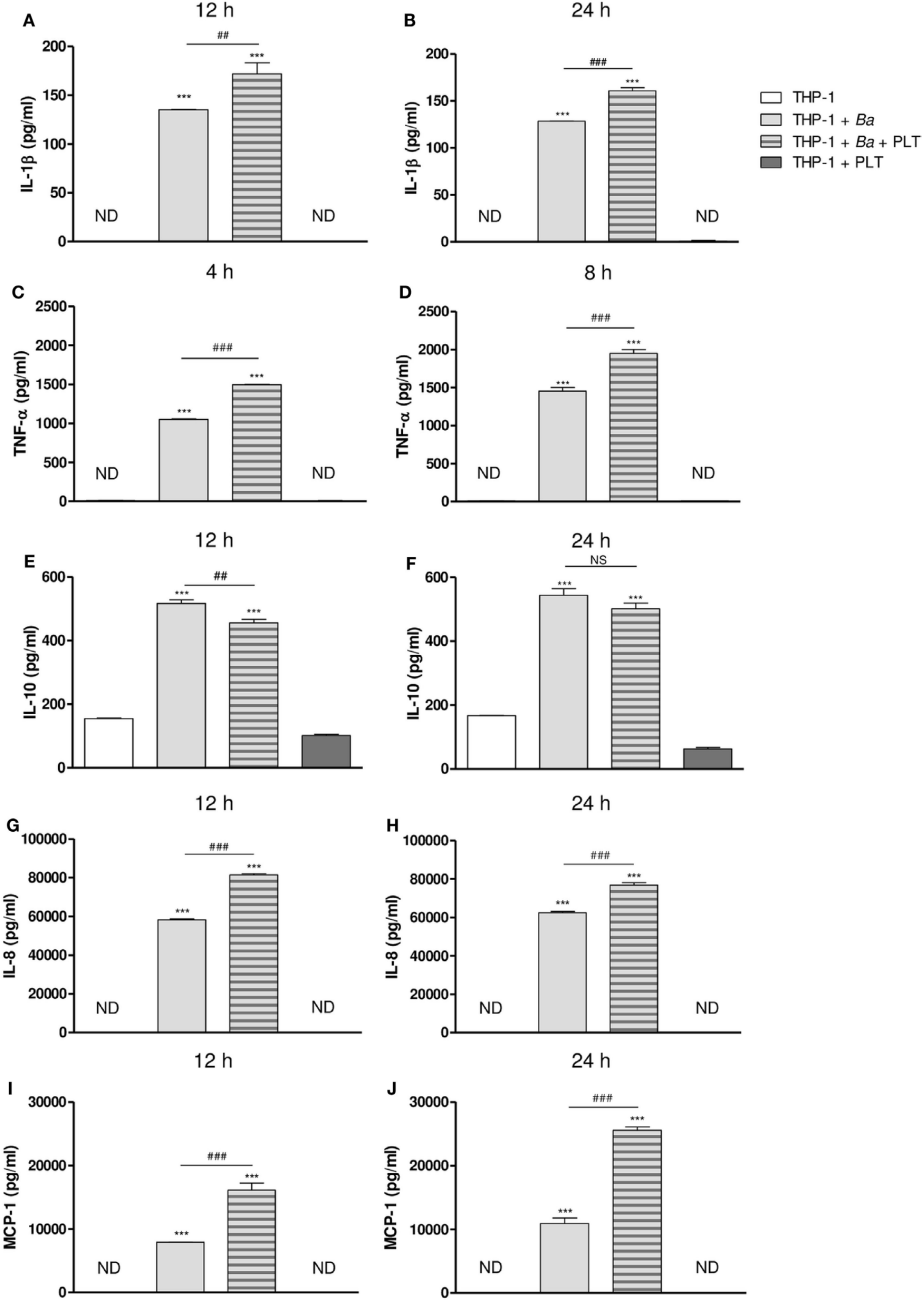
Platelets modulate cytokine and chemokine secretion in the context of *Brucella abortus* infection. THP-1 cells were infected with *B. abortus* in presence or absence of platelets for 4, 8, 12, and 24 h, as indicated. Supernatants were then collected and IL-1β **(A,B)**, TNF-α **(C,D)**, IL-10 **(E,F)**, IL-8 **(G,H)**, and MCP-1 **(I,J)** concentration was quantified by ELISA. ***P < 0.001 vs. THP-1. ^##^*P* < 0.01; ^###^*P* < 0.001 vs. THP-1 + *Ba*. NS, not significant; ND, not detected. THP-1:PTL:*Ba* 1:100:100.

After studying the interaction among monocytes, *B. abortus*, and platelets, we decided to evaluate the ability of platelets to modulate functional aspects of monocytes/macrophages. Particularly, we focused on the immunomodulatory cytokine/chemokine secretion, a key aspect of immunity against bacterial infections. For this, THP-1 cells were infected with *B. abortus* in presence or absence of platelets for different time periods. Then, supernatants were collected and the secretion of different cytokines and chemokines was quantified by ELISA. *B. abortus* infection increased the secretion of IL-1β, TNF-α, IL-10, IL-8, and MCP-1. The presence of platelets during monocyte infection enhanced the secretion of the pro-inflammatory cytokines IL-1β and TNF-α (Figures 6A–D), and the chemokines IL-8 and MCP-1 at both analyzed times (Figures 6G–J). Conversely, the presence of platelets in the context of infection decreased the secretion of the anti-inflammatory cytokine IL-10 (Figures 6E,F). Platelets alone or in presence of *B. abortus* did not secrete detectable levels of the cytokines/chemokines studied (data not shown). These results demonstrate that platelets potentiate the secretion of pro-inflammatory cytokines and chemokines by *B. abortus*-infected monocytes.

### Platelets Modulate Adhesion and Co-Stimulatory Molecules Expression on Monocytes in the Context of *B. abortus* Infection

**Figure 7:**
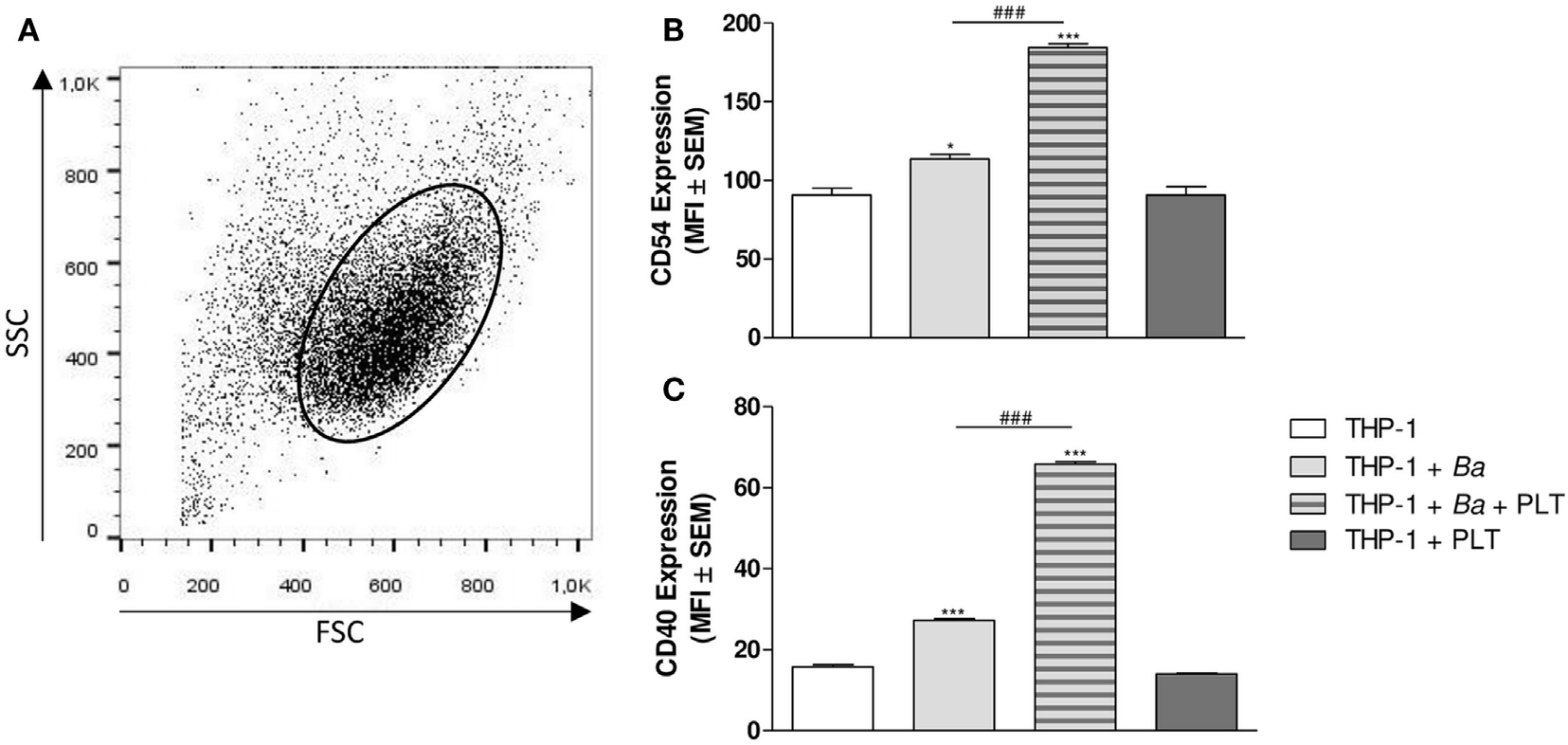
Platelets modulate adhesion and co-stimulatory molecules expression on monocytes in the context of *Brucella abortus* infection. THP-1 cells were infected with *B. abortus* in presence or absence of platelets for 4 h. **(A)** Monocyte population was identified in a FSC vs. SSC dot plot. The surface expression of CD54 **(B)** and CD40 **(C)** was assessed by flow cytometry within this region. Bars represent the arithmetic means ± SEM of five experiments. MFI, mean fluorescence intensity. *P < 0.05; ***P < 0.001 vs. THP-1. ^###^*P* < 0.001 vs. THP-1 + *Ba*. THP-1:PTL:*Ba* 1:100:100.

Under inflammatory conditions, monocytes express a great amount of adhesion molecules, among which CD54 (ICAM-1) is found (37). In fact, CD54 has been involved in leukocytes complexes formation and in the adhesion to the endothelium (38). On the other hand, these activated monocytes increase the expression of co-stimulatory molecules on their surface, such as CD40, facilitating the antigen presentation to cells of adaptive immunity (39). Therefore, we next focused on the ability of platelets to modulate the expression of CD54 and CD40 on monocytes. For this, THP-1 cells were infected with *B. abortus* in presence or absence of platelets for 4 h. Then, the surface expression of CD54 and CD40 within the monocyte gate was determined by flow cytometry (Figure 7A). *B. abortus* infection slightly but significantly increased the expression of CD54 and CD40 (Figures 7B,C). Nevertheless, the presence of platelets during *B. abortus* infection enhanced both CD54 and CD40 expression on infected monocytes (Figures 7B,C). Overall, these results indicate that platelets not only establish complexes with *B. abortus*-infected monocytes, but they also modulate cytokine/chemokine secretion and co-stimulatory/adhesion molecules expression of these monocytes, enhancing their pro-inflammatory capacity.

### Soluble Factors Released by Platelets Mediate the Modulation of Surface Molecules on Monocytes

**Figure 8:**
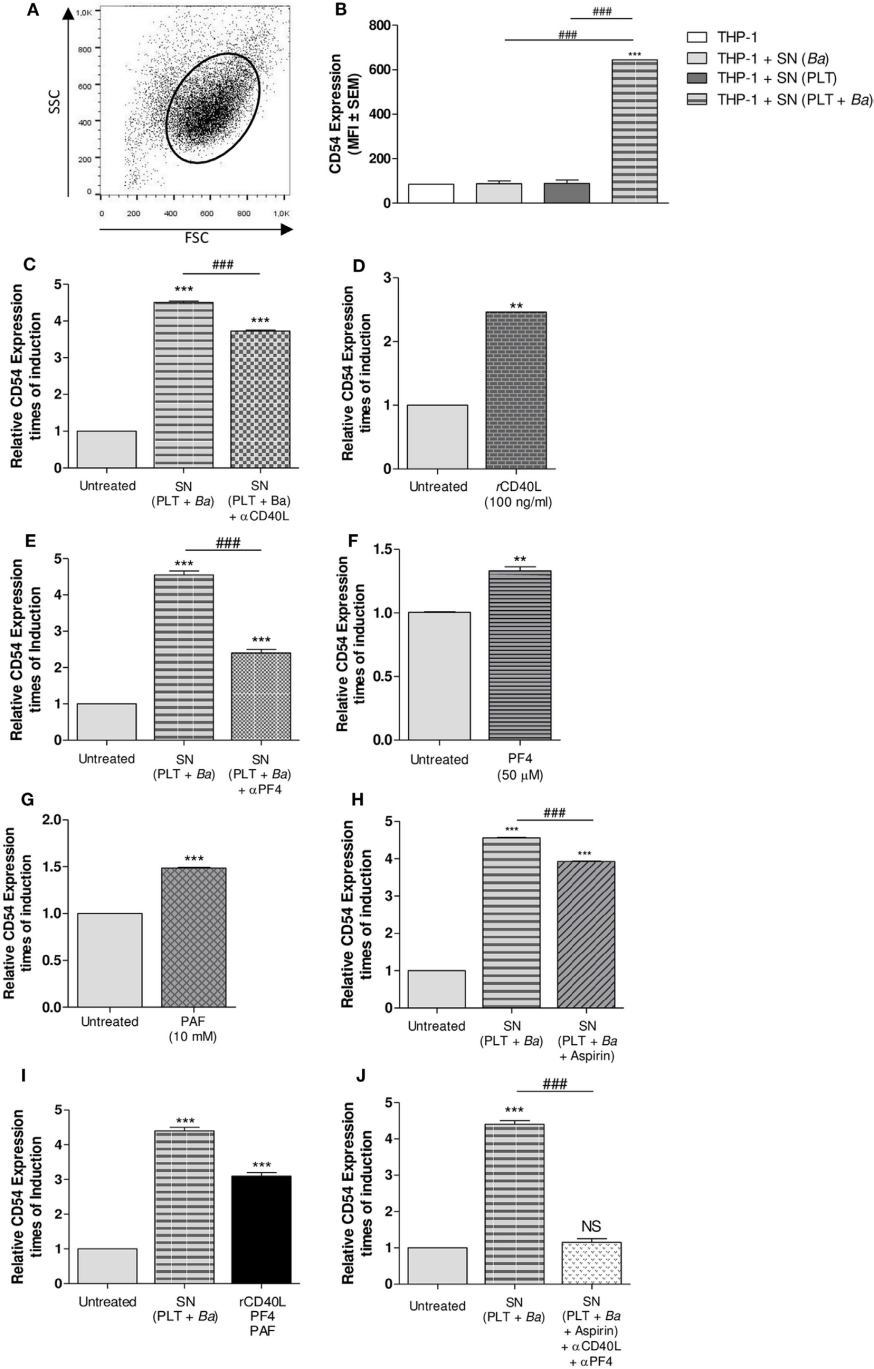
Soluble factors released by platelets mediate the modulation of surface molecules on monocytes. THP-1 cells were stimulated with supernatants collected from platelets incubated in presence or absence of *Brucella abortus* and assessed by flow cytometry. Monocyte population was identified in a FSC vs. SSC dot plot **(A)** and the CD54 surface expression was quantified within this region **(B)**. Supernatants from bacteria cultured alone were used as control. Bars represent the arithmetic means ± SEM of three experiments. MFI, mean fluorescence intensity. THP-1 cells were incubated with supernatants of *B. abortus*-stimulated platelets in presence or absence of neutralizing Ab to soluble CD40L (sCD40L) **(C)** or platelet factor 4 (PF4) **(E)**. THP-1 cells were stimulated with sCD40L **(D)**, PF4 **(F)**, or platelet-activating factor (PAF) **(G)** in their recombinant forms. **(H)** THP-1 cells were treated with supernatant collected from *B. abortus*-stimulated platelets in presence or absence of aspirin. **(I)** THP-1 cells were stimulated with a combination of sCD40L, PF4, and PAF. **(J)** THP-1 cells were treated with supernatant obtained from *B. abortus*-stimulated platelets in presence or absence of aspirin plus anti-sCD40L and anti-PF4 neutralizing antibodies. In all cases, CD54 surface expression was assessed by flow cytometry after the different treatments. Bars represent the times of CD54 induction ± SEM of three experiments. **P < 0.01; ***P < 0.001 vs. untreated. ^###^*P* < 0.001. NS, not significant. THP-1:PTL:*Ba* 1:100:100.

We next wondered whether the modulation of monocytes by platelets in the context of *B. abortus* infection required physical contact between these cell populations; and/or it was mediated by soluble factors. To answer this question, THP-1 cells were stimulated with supernatants collected from platelets incubated in presence or absence of *B. abortus*. THP-1 cells were also stimulated with supernatants collected from bacteria cultured alone as control. Then, the surface expression of CD54 within the monocyte gate was determined by flow cytometry (Figure 8A). Culture supernatant from platelets incubated in presence of *B. abortus* was able to induce the expression of CD54 on monocytes, unlike those of platelets or bacteria alone (Figure 8B). This result indicates that there are soluble factors involved in the monocyte modulation and that physical contact between platelets and monocytes is not indispensable for the modulation of, at least, this surface molecule. Among several mediators released by platelets upon activation, sCD40L, PF4, PAF, and TXA_2_ have been previously involved in the formation of platelet–monocyte complexes in numerous infectious diseases (40–42). Therefore, we evaluated the possible role of these factors in the modulation of *B. abortus*-infected monocytes mediated by platelets. For this, the expression of CD54 on monocytes was evaluated in neutralization experiments and/or in experiments using recombinant proteins. First, THP-1 cells were incubated with supernatants of *B. abortus*-stimulated platelets in presence or absence of an anti-sCD40L neutralizing Ab. Neutralization of sCD40L resulted in a partial reversion of the CD54 induction mediated by the supernatant of *B. abortus*-stimulated platelets (Figure 8C). Confirming this result, exposure of THP-1 to recombinant sCD40L slightly but significantly induced the surface expression of CD54 on monocytes (Figure 8D). Next, THP-1 cells were cultured with supernatants of *B. abortus*-stimulated platelets in presence or absence of an anti-PF4 neutralizing Ab. Neutralization of PF4 was able to partially reverse the induction of CD54 expression mediated by the supernatant of *B. abortus*-stimulated platelets (Figure 8E). According with this result, recombinant PF4 was able to slightly induce the expression of CD54 on monocytes (Figure 8F). To evaluate the role of PAF, THP-1 cells were incubated with the recombinant form of this mediator. Exposure of THP-1 to recombinant PAF slightly but significantly induced the expression of CD54 on monocytes (Figure 8G). Finally, regarding the role of TXA_2_, THP-1 cells were treated with supernatant collected from *B. abortus*-stimulated platelets in presence or absence of the cyclooxygenase inhibitor aspirin. The inhibition of TXA_2_ formation partially reversed the induction of CD54 expression mediated by the supernatant of *B. abortus*-stimulated platelets (Figure 8H). Furthermore, the induction of CD54 by the combination of sCD40L, PF4, and PAF was more potent than the induction obtained by each recombinant protein alone (Figure 8I). In addition, supernatant collected from *B. abortus*-stimulated platelets in presence of aspirin and then neutralized by anti-sCD40L and anti-PF4 was able to completely reverse the induction of CD54 expression (Figure 8J). Overall, these results demonstrate that there is not a unique soluble factor involved in the CD54 modulation of *B. abortus*-infected monocytes mediated by platelets. On the contrary, several factors such as sCD40L, PF4, PAF, and TXA_2_ partially and simultaneously contribute to this phenomenon.

### Platelets Promote Monocyte Invasion by *B. abortus* but Improve Its Control

**Figure 9:**
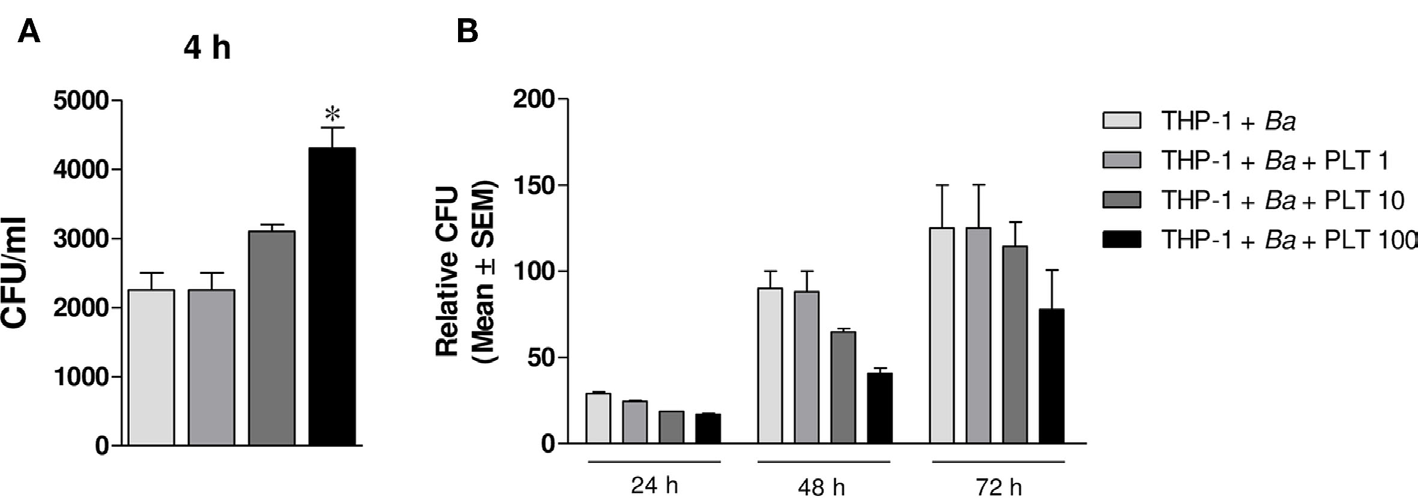
Platelets promote monocyte invasion by *Brucella abortus* but improve its control. THP-1 cells were infected with *B. abortus* in presence or absence of platelets at different ratios (THP-1:PLT 1:1, 1:10, and 1:100) for 4 h and then incubated with antibiotics for 24, 48, or 72 h. **(A)** Quantification of monocyte invasion by *B. abortus* after 4 h. Data are expressed as colony-forming units (CFU) per ml. **(B)** Quantification of monocyte invasion by *B. abortus* at different times post-infection. Data are expressed as relative CFU calculated as: CFU per ml at each time point (24, 48, or 72 h) relative to the CFU per ml measured for the same condition at 4 h.

Finally, we wondered whether the presence of platelets modulate the time course of *B. abortus* infection. For this, THP-1 cells were infected with *B. abortus* in presence or absence of platelets at different ratios for 4 h. Then, extracellular bacteria were killed by adding antibiotics and the cells were incubated for 24, 48, or 72 h to study the time course of the infection. Monocytes were finally lysed, and the number of viable intracellular bacteria was determined by plating the lysates on tryptose-soy agar. As shown in Figure 9A, and in agreement with the results shown in Figure 2, the presence of platelets significantly increased the percentage of *B. abortus*-infected THP-1 cells. Nevertheless, the presence of platelets subsequently improved the control of the infection at longer times (Figure 9B). This result demonstrates that despite promoting the invasion of monocytes, platelets improve the control of *B. abortus* within the infected monocytes.

## Discussion

This is the first study elucidating the role of platelets in the immune response against *B. abortus*. Particularly, we demonstrated that platelets directly interact with *B. abortus* and behave as carriers of these bacteria promoting the invasion of monocytes/macrophages. As a consequence of this activity, platelets establish complexes with infected monocytes. This platelet–monocyte interaction, together with the release of soluble factors, triggers an increase in the pro-inflammatory response of the infected monocytes.

Our interaction experiments between *B. abortus* and platelets demonstrated that both populations are intimately associated. Moreover, this interaction is capable of triggering platelet activation. In line with this result, it has been described that platelet activation can be triggered by bacteria binding to TLRs, receptors for the complement, FcγRII, and/or several adhesion molecules present on platelets surface, as demonstrated for *Streptococcus sanguinis, Staphylococcus epidermidis*, and *Chlamydia pneumoniae* (36).

Interestingly, our results indicated that bacteria not only bind to platelets but also invade them. These results are in agreement with several studies which demonstrate the ability of activated platelets to internalize bacteria such as *Staphylococcus aureus, Escherichia coli*, and *Porphyromonas gingivalis* (43–46). These studies suggest that internalization might have consequences in bacterial fate. It would either protect the bacteria from the immune system or promote the lysis of the bacteria within the platelet cytoplasm (43–46). Although the presence of phagolysosomes in platelets has not been demonstrated yet, it has been observed that platelets can actively phagocyte *S. aureus* and secrete antimicrobial molecules such as β-defensin. In turn, this leads to the formation of platelet–neutrophil complexes and NETs which help in the bacteria immobilization and elimination (24). The fact that an intracellular pathogen such as *B. abortus* invades a short half-life cell population might seem curious, taking into account the chronic predisposition of this infection. However, it is possible that the internalization of the bacteria by platelets functions as a mechanism to evade the immune system. Thus, *B. abortus* would remain hidden inside platelets until it reaches its main replicative niche, the monocyte/macrophage. In line with this, our result showed that platelets behave as carriers of *B. abortus* toward the monocytes and thus they might help with the transportation of the bacteria through the bloodstream to its replicative niche. Nevertheless, whether the bacteria may also be destroyed inside platelets remains unknown and it is an interesting issue which merits further investigation.

The central role of monocytes/macrophages has been extensively described in *Brucella* infection. During the initial stages of infection, macrophages show a significant microbicidal capacity, contributing to the control of the infection. However, they have also been proved to be the main niche for *B. abortus* replication, allowing the persistence of the bacteria. Therefore, once the interaction between *B. abortus* and platelets was corroborated, we focused on investigating whether platelets were able to modulate the infective capacity and/or immunological features of these cells. Our results demonstrated that the presence of platelets promote *B. abortus*-mediated invasion of monocytes/macrophages. In addition, our results showed that the co-incubation of the three cell populations leads to the formation of platelet–monocyte complexes. Moreover, the disposition of platelets in these complexes was particular, as they completely surround the infected monocytes creating rosettes as has been described in the literature (47). In line with our results, platelets have been widely implicated in the formation of complexes with neutrophils and monocytes (12–14). It has been described that the interaction between platelets and monocytes is predominantly through the interaction of P-selectin with the ligand of P-selectin (PSGL-1) expressed on the monocyte surface (48, 49). It has been proven that the interaction of activated platelets with monocytes induce the maturation of a pro-inflammatory subtype of monocytes (47, 50). In addition, these complexes are usually increased in the circulation of patients with chronic inflammation-associated diseases, such as HIV (41, 42). Taking together, these observations suggest that the presence of platelet–monocyte complexes in the bloodstream may be used as an indicator of an inflammatory state.

Based on our confocal microscopy studies, we were able to demonstrate that platelets form complexes with *B. abortus*-infected monocytes. Furthermore, the interaction between platelets and bacteria triggers platelet activation, promotes the transportation of bacteria toward monocytes and, as a consequence, the formation of platelet–monocyte complexes. In line with these results, other *in vitro* studies have shown that platelet activation, rather than monocyte activation, is responsible for the formation of these mixed complexes (51).

Regarding the modulation of functional aspects of monocytes/macrophages, our results demonstrated that the presence of platelets in the context of *B. abortus* infection increases the secretion of IL-1β, TNF-α, IL-8, and MCP-1, while it inhibits the secretion of IL-10. Supporting these results, it has been described that the interaction between monocytes and platelets for prolonged time induces an increase in the secretion of IL-8, MCP-1, and IL-1β (47, 52) together with other soluble factors such as tissue factor (47, 53–55). In particular, it has been proven that in the context of an *S. aureus*-mediated infection, activated platelets release β-defensin which slow down the bacterial growth rate, induces the formation of NETs and the expression of IL-8 and MCP-1 by macrophages (24).

With respect to the modulation of surface molecules, our results demonstrated that the presence of platelets increases the expression of the intercellular adhesion molecule CD54 (ICAM-1). These results, together with the increase in MCP-1 secretion, suggest the presence of a positive feedback loop, which promote the recruitment and adhesion of circulating monocytes to the activated endothelium in infected foci.

Although both P-selectin and GPIIb/IIIa are usually involved in the intracellular signaling pathways activated in the platelet–leukocyte crosstalk, Li et al. have suggested that this crosstalk would be mainly dependent on soluble mediators, and not on cell–cell interactions (51). In line with this suggestion, our results demonstrated that platelets are able to modulate *B. abortus*-infected monocytes functionality, at least in part, by secretion of soluble factors. Particularly, we confirmed that sCD40L, PF4, PAF, and thromboxane A2 released from platelets orchestrate the CD54 modulation on monocytes. According to these results, it has been proposed that platelet-derived sCD40L modulates the activation state of monocytes, by interacting with CD40 in the monocyte surface. In particular, this interaction has been shown to stimulate the secretion of cytokines, such as IL-8, and the monocytes tumoricidal activity, without additional stimulus (39). Supporting the importance of the sCD40L–CD40 interaction, our results demonstrated that platelets were also able to increase the expression of CD40 on the surface of monocytes.

It has been recently demonstrated that PF4 and thromboxane A2 released by platelets are soluble mediators involved in the formation of NETs (56). Moreover, PF4 prevents the spontaneous apoptosis of human monocytes and induces the differentiation of monocytes into a specific subtype of macrophages (57). In addition, PF4 is a potent activator of phagocytosis and promotes the respiratory burst in human monocytes as well as human and murine macrophages (58).

Regarding PAF, it has been recently demonstrated that PAF receptor (PAFR) activation promotes phagocytosis, and it is involved in the uptake of *B. abortus* by monocytes (59). However, in this study, the authors only used recombinant PAF to stimulate monocytes without taking into account the role of platelets during infection. In light of our experiments, we might speculate that PAF released by platelets, through their binding to the PAFR, would lead to the greater invasion of monocytes by *B. abortus* that we observed in the presence of platelets.

The role of platelets in the pathogenesis of diverse infectious diseases is a topic worth of discussion. Recently, it has been described that platelets contribute to *Streptococcus pyogenes* dissemination (12). In addition, mice infected with these bacteria exhibited thrombocytopenia, platelet activation, and formation of platelet–neutrophil complexes. In this context, platelet-depleted mice showed reduced bacterial dissemination rate and decreased levels of systemic IL-6 (12). By contrast, other studies reveal that platelet-depleted mice rapidly succumb to LPS- (60) or *S. aureus*-induced sepsis (14). Likewise, it has been demonstrated *in vivo* that the interaction between platelets and Kupffer cells in liver contribute to blood-borne pathogens elimination such as *Bacillus cereus* and *S. aureus* (61). Thus, platelets can play either a beneficial or detrimental role during the infection elicited by different pathogens. But what is the role of platelets during the development of human brucellosis? Our result demonstrates that platelets promote the invasion of monocytes by *B. abortus*. Moreover, platelets potentiate the secretion of monocyte-derived pro-inflammatory cytokines and chemokines, causing a pro-inflammatory environment. This microenvironment, along with direct interaction within platelet–monocyte complexes, may induce monocytes differentiation into a potent pro-inflammatory profile with an enhanced microbicidal capacity. Altogether, these results suggest a protective role of platelets in brucellosis in which platelets diminish the bacterial load in the bloodstream by promoting the uptake and control of *B. abortus* by monocytes. Nevertheless, bacteria are able to evade the early defense mechanisms establishing a chronic infection. In fact, it has been described that patients with chronic brucellosis usually presents thrombocytopenia, which is associated with greater severity of the disease (4, 6). Many causes could explain the decreased platelet count observed in patients with brucellosis. Our results suggest that the formation of platelet–monocyte complexes could contribute, at least in part, to decreasing the level of platelets in the bloodstream. In line with this, it was demonstrated that the activation of platelets and the formation of platelet–monocyte aggregates contribute to the decreased platelet count observed in macaques during the infection with acute simian immunodeficiency virus (62). The causes that determine the brucellosis-associated thrombocytopenia and the *in vivo* role of platelets during the development of this disease are important aspects that remain to be explored.

Given the potential protective role of platelets, we postulate that the reduction of platelets in the bloodstream, due to the formation of platelet–monocyte complexes, could contribute to the development of brucellosis and the persistence of infection. Overall, our results highlight the relevance of platelets as contributors to host defense against *Brucella*.

## Ethics Statement

Human platelets and monocytes were isolated exclusively from healthy adult blood donors in agreement with the guidelines of the Ethical Committee of the IMEX Institute (protocol number: 20160518-M). All adult blood donors provided their informed consent prior to the study in accordance with the Declaration of Helsinki (2013) of the World Medical Association.

## Author Contributions

AT, LV, RP, and PB conceived and designed the experiments. AT, LV, MM, MD, AR, and VIL performed the experiments. AT analyzed the data and wrote sections of the manuscript. MD performed the infections with viable *B. abortus*. GG and RP supported the work with key suggestions and helped with data interpretation. PB supervised experiments, interpreted the data, and wrote the manuscript. All authors reviewed the manuscript.

## Conflict of Interest Statement

The authors declare that the research was conducted in the absence of any commercial or financial relationships that could be construed as a potential conflict of interest.

## Supplementary Material

Video S1 Platelets completely surround *B. abortus*-infected monocytes.

Video S2 Platelets pre-treated with *B. abortus* completely surround monocytes.

Video S3 Platelets deliver bacteria toward monocytes.
