# Supplementary figures and images for: Platelets Promote Brucella abortus Monocyte Invasion by Establishing Complexes With Monocytes
Source: Front Immunol. 2018 May 7;9:1000. doi: 10.3389/fimmu.2018.01000 (PMC5949576; doi:10.3389/fimmu.2018.01000)

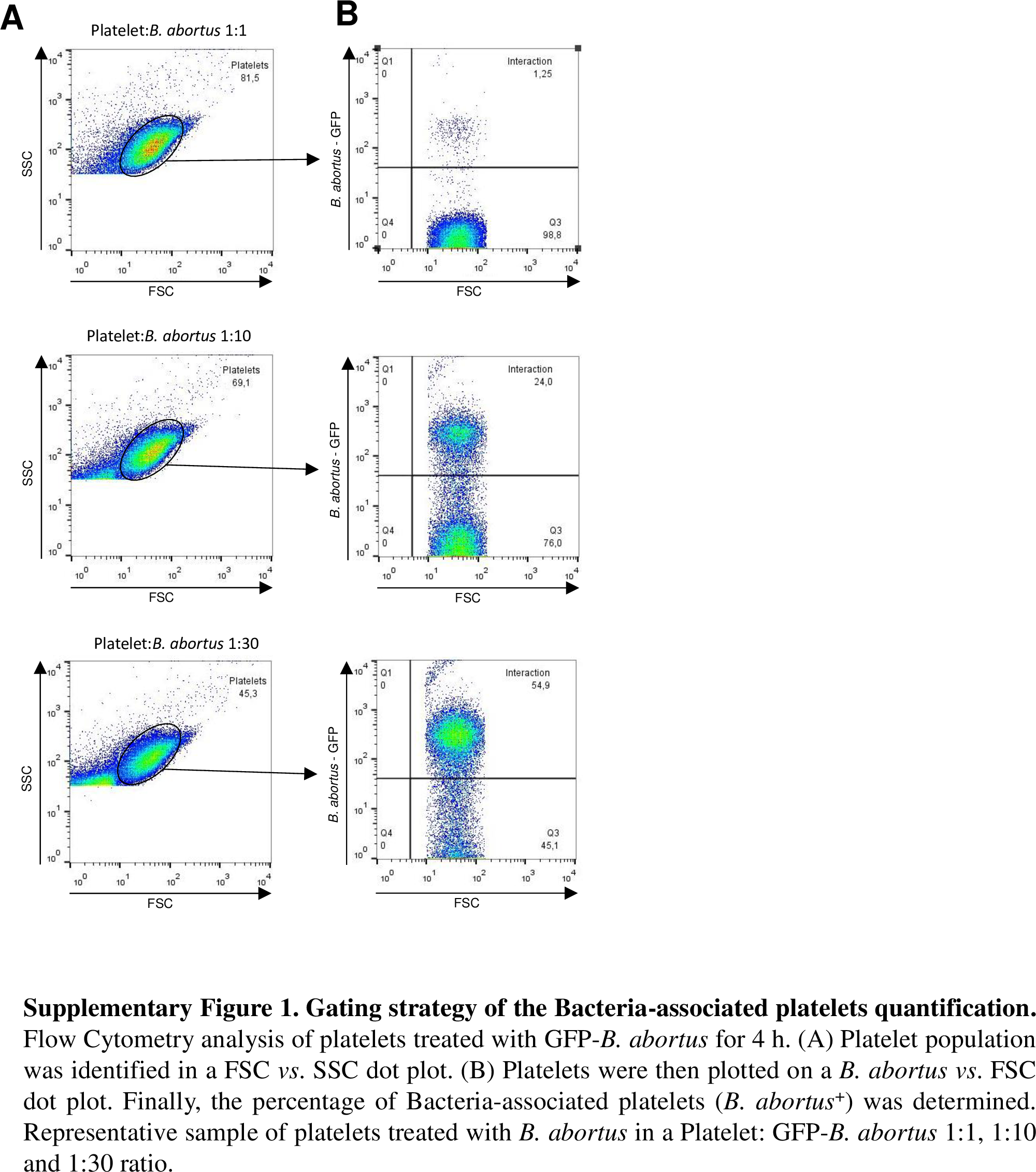

Supplement: Supplementary file 4 [file Image_1.TIF]
